# Identification of Candidate Genes and Genomic Selection for Seed Protein in Soybean Breeding Pipeline

**DOI:** 10.3389/fpls.2022.882732

**Published:** 2022-06-16

**Authors:** Jun Qin, Fengmin Wang, Qingsong Zhao, Ainong Shi, Tiantian Zhao, Qijian Song, Waltram Ravelombola, Hongzhou An, Long Yan, Chunyan Yang, Mengchen Zhang

**Affiliations:** ^1^National Soybean Improvement Center Shijiazhuang Sub-Center, North China Key Laboratory of Biology and Genetic Improvement of Soybean, Ministry of Agriculture, Hebei Laboratory of Crop Genetics and Breeding, Cereal & Oil Crop Institute, Hebei Academy of Agricultural and Forestry Sciences, Shijiazhuang, China; ^2^Department of Horticulture, University of Arkansas, Fayetteville, AR, United States; ^3^Soybean Genomics and Improvement Lab, United States Department of Agriculture - Agricultural Research Service (USDA-ARS), Beltsville, MD, United States; ^4^Department of Soil and Crop Sciences, Texas A&M University, College Station, TX, United States

**Keywords:** *Glycine max*, genome-wide association study, genomic selection, genotyping by sequencing, protein content, single nucleotide polymorphism

## Abstract

Soybean is a primary meal protein for human consumption, poultry, and livestock feed. In this study, quantitative trait locus (QTL) controlling protein content was explored *via* genome-wide association studies (GWAS) and linkage mapping approaches based on 284 soybean accessions and 180 recombinant inbred lines (RILs), respectively, which were evaluated for protein content for 4 years. A total of 22 single nucleotide polymorphisms (SNPs) associated with protein content were detected using mixed linear model (MLM) and general linear model (GLM) methods in Tassel and 5 QTLs using Bayesian interval mapping (IM), single-trait multiple interval mapping (SMIM), single-trait composite interval mapping maximum likelihood estimation (SMLE), and single marker regression (SMR) models in Q-Gene and IciMapping. Major QTLs were detected on chromosomes 6 and 20 in both populations. The new QTL genomic region on chromosome 6 (Chr6_18844283–19315351) included 7 candidate genes and the Hap.X*^AA^* at the Chr6_19172961 position was associated with high protein content. Genomic selection (GS) of protein content was performed using Bayesian Lasso (BL) and ridge regression best linear unbiased prediction (rrBULP) based on all the SNPs and the SNPs significantly associated with protein content resulted from GWAS. The results showed that BL and rrBLUP performed similarly; GS accuracy was dependent on the SNP set and training population size. GS efficiency was higher for the SNPs derived from GWAS than random SNPs and reached a plateau when the number of markers was >2,000. The SNP markers identified in this study and other information were essential in establishing an efficient marker-assisted selection (MAS) and GS pipelines for improving soybean protein content.

## Introduction

Soybean [*Glycine max* (L.) Merr.] provides about 60% of the vegetable-derived proteins worldwide and is a primary meal protein for human consumption, poultry, and livestock feed (Wolf, 1970; Patil et al., 2017). Improving protein content is one of the major breeding objectives in breeding programs (Li S. et al., 2019; Stewart-Brown et al., 2019). Traditional soybean breeding methods require phenotyping and multigeneration selection. Although molecular marker-assisted selection (MAS) by tagging the desired genes during breeding selection is an approach to make the selection more efficient (Collard et al., 2005), it is only relatively effective for traits with high heritability and controlled by major genes (Xu and Crouch, 2008; Xu et al., 2012; Patil et al., 2017). Genomic selection (GS) was developed for the selection of traits controlled by multiple genes, but it has not been practically applied due to the large variation of prediction accuracy in different populations and lacking efficient genotyping platforms (Zhang A. et al., 2017; Liu et al., 2018). With the rapid development of genomic tools and DNA sequencing technology, breeders and geneticists are able to explore molecular approaches to increase seed protein genetic gain (Song et al., 2004, 2013; Schmutz et al., 2010; Wang et al., 2020).

Linkage analysis (Hyten et al., 2004; Nichols et al., 2006; Pathan et al., 2013; Teng et al., 2017; Whiting et al., 2020) and genome-wide association study (GWAS) are powerful tools to identify markers associated with seed protein content in soybean (Hwang et al., 2014; Leamy et al., 2017; Lee et al., 2019; Li S. et al., 2019); to date, a total of 262 loci have been reported through linkage analysis and 107 loci have been reported through GWAS (Patil et al., 2017; Gangurde et al., 2020) per SoyBase.^1^ These loci were on all the chromosomes, especially chromosome (Chr.) 15 and Chr. 20 (see text footnote 1/). Among these, several quantitative trait loci (QTLs), such as *cqPro-20* on Chr. 20 and *cqPro-15* on Chr. 15, were confirmed based on a low error rate (lower than 0.01) and in different populations (Patil et al., 2017). More than 150 candidate genes have been suggested to control seed protein content in soybean (Zhang D. et al., 2017; Zhang J. et al., 2018; Zhang Y. et al., 2018; Li S. et al., 2019; Zhang et al., 2019; Wang et al., 2020). The most described genes affecting seed protein content were sugar efflux transporter SWEET39 (*Glyma15g05470*) and sugar efflux transporter SWEET24 (*Glyma08g19580*) (Wang et al., 2020).

The populations used for mapping protein content in the previous reports included pedigree-based F2 and F4:6 (Csanádi et al., 2001; Chapman et al., 2003), recombinant inbred lines (RILs) population (Qi et al., 2014; Hacisalihoglu et al., 2018), backcross population (Sebolt et al., 2000; Liang et al., 2010), multiline population (Brummer et al., 1997; Wang et al., 2014; Whiting et al., 2020), nested association mapping population (Gangurde et al., 2020), and natural population (Hwang et al., 2014; Bandillo et al., 2015; Li D. et al., 2019). Most studies used a single population, but some studies used two populations for QTL verification (Vaughn et al., 2014; Zhang D. et al., 2017; Zhang et al., 2019); a few studies analyzed QTL using both the linkage mapping and associate mapping methods (Zhang et al., 2019).

The annual wild soybean (*Glycine soja*) is an important resource to improve soybean (Lam et al., 2010; Yao et al., 2020). Therefore, the objectives of this study were to: (1) identify QTL conferring seed protein content in RILs derived from cultivated and wild soybeans; (2) identify single nucleotide polymorphism (SNP) markers associated with seed protein content in GWAS and candidate genes controlling the trait; and (3) assess the accuracy of GS base on different SNP sets, training population size, and statistical models.

## Materials and Methods

### Plant Materials

#### Recombinant Inbred Line

A population of 180 F9-derived RILs was developed from a cross of Jidou12 (*Glycine max*) and Ye9 (*Glycine soja*). Jidou12 is a high-yield cultivar with a high protein content that is grown in Shandong Jiaodong Peninsula, Hebei Province, and south-central Shanxi. The seed protein content averaged 46.48% for Jidou12 and 48.78% for Ye9 on a dry weight basis.

#### Natural Population

A total of 284 soybean genotypes, including 250 accessions selected from germplasm collection by Dr. Lijuan Qiu’s laboratory at the Chinese Academy of Agricultural Sciences and 34 cultivars from Hebei Province, were used for the protein association analysis (Supplementary Table 1). These genotypes were originally from 10 provinces in China (202, 67.5%) and 6 states in the United States (76, 30.1%), South Korea (3, 1.2%), and Japan (2, 0.8%).

### Field Design

Field experiments were conducted at Shijiazhuang (114°83′E, 38°03′N) in Hebei Province in a randomized complete block design with three replications in 2008, 2010, 2019, and 2020. The plot size was 3 m × 6 m with six rows and 50 cm space between rows in all the trials. The planting density was 225,000 plants per ha. Each year, the plots were irrigated once at the seed-filling stage. Plants were harvested after 95% of the leaves were falling off. Ten plants were randomly chosen from the middle of the plot for indoor laboratory seed protein content analysis when 95% of plants in the plot were matured.

### Statistical Analysis of Phenotypic Data

Seed protein content was quantified using Fourier transform-near IR spectroscopy (Bruker MPA, Karlsruhe, Germany) at the North China Key Laboratory of Biology and Genetic Improvement of Soybean, Ministry of Agriculture. Under the Quant 2 method of OPUS (https://www.bruker.com/en/products-and-solutions/infrared-and-raman/opus-spectroscopy-software/downloads.html) version 5.5 software (Bruker MPA, Karlsruhe, Germany), the samples’ protein content data were calculated using the dry basis model (Yan et al., 2008). Each RIL and accession from each replication of each environment were detected three times using about 100–150 dry seeds and the average was used for statistical analysis. Analysis of variance was performed using JMP^®^ (https://www.jmp.com/en_us/home.html) Genomics 7 (Sall et al., 2017). The least-squares mean (LSM) of the protein content of each soybean genotype from JMP was used as the phenotypic data in the association mapping.

### Genotyping by Sequencing and Single Nucleotide Polymorphism Discovery

Genomic DNA was extracted from leaves of soybean plants using the QIAGEN DNeasy Plant Mini Kit (250). DNA was digested using the restriction enzyme *Ape*KI following the genotyping by sequencing (GBS) protocol described by Elshire et al. (2011). The 90 bp pair-end sequencing of accessions was performed using an Illumina HiSeq 2000 machine at the Genetic Research Institute, Chinese Academy of Sciences. GBS data alignment, mapping, and SNP discovery were done using Short oligonucleotide analysis Package (SOAP) family software. An average of 3.26 M short reads for each accession was aligned to soybean whole-genome sequence (Wm82.a2.v1) using SOAPaligner/soap2. SOAPsnp version 1.05 was used for SNP calling (Li et al., 2009; Li, 2011). Approximately, a half-million SNPs were discovered among the 284 soybean germplasm accessions. The SNPs were filtered before genetic diversity and association analyses. Soybean accession with >5% missing SNP and the >2% heterozygous SNP genotypes was eliminated. After the SNP dataset was filtered to remove those SNPs with minor allele frequency (MAF) <2%, missing data >5%, and heterozygous genotype >25%, a total of 10,115 SNPs were used for genetic diversity and association analysis (Supplementary Figure 1).

### Genetic Maps

The genetic maps were constructed with JoinMap 4.0 (Van Ooijen, 2006) when the threshold for the logarithm of odds (LOD) was 3.0 based on 180 F9 RILs. QTL analysis of protein content in the RIL population was performed using single-trait Bayesian interval mapping (BIM), single-trait multiple interval mapping (SMIM), single-trait composite interval mapping maximum likelihood estimator (SMLE), single marker regression (SMR) method of Q-gene software (Joehanes and Nelson, 2008) with inclusive composite interval mapping (ICIM, http://www.isbreeding.net) (Meng et al., 2015). Variance components, QTL heritability, and QTL effect for seed protein content were estimated by QTLNetwork version 2.1 based on the phenotypic data (Yang et al., 2008). Only the QTL, which was mapped in similar physical locations (<1,500 kb) on the same chromosomes based on the five methods, was defined as a reliable QTL. The selected SNP markers were further tested for their effect by variance analysis using JMP Pro 10 (Sall et al., 2017).

### Population Genetic Diversity and Association Analysis

STRUCTURE is a program that uses Bayesian methods to analyze multilocus data in population genetics (Kaeuffer et al., 2007). This study used a hybrid model and an allelic variation occurrence non-correlative model to examine the population structure of soybean germplasm. The number of the subpopulation (K) was assumed to be between 1 and 12. Each K was run 10 times, the Markov Chain Monte Carlo (MCMC) length of the burn-in period was 20,000, and the number of the MCMC iterations after the burn-in was 50,000. Delta K was used to determine appropriate *K*-values (Earl and vonHoldt, 2012). Next, CLUMPP was used to integrate the STRUCTURE-generated results with the “repeat 1,000” parameter. In addition, two different association mapping models were used to analyze the association between the molecular markers and traits, the TASSEL general linear model (GLM-Q), and the mixed linear model (MLM) combining kinship with population structure (Q-matrix) (Yu et al., 2006; Bradbury et al., 2007).

### Identification of Candidate Genes

Linkage disequilibrium (LD) analysis was performed in the regions with SNP significantly associated with protein content; SNPs with *r*^2^ > 0.5 in a 1-Mb window were considered to be in one linkage disequilibrium (LD) block in the heterochromatic regions. Haplotype analysis was conducted on all the SNPs within the LD block containing significant loci. Two databases, namely, the SoyBase(see text footnote 1) and the *Arabidopsis* Information Resource,^2^ were used for gene annotation and preliminary screening of candidate genes were determined by combined bioinformatics and statistics.

### Genomic Selection

Ridge regression best linear unbiased prediction (rrBLUP) and Bayesian Lasso Regression (BLR) were used to predict genomic estimated breeding value (GEBV) in GS (Endelman, 2011; Legarra et al., 2011). The packages “rrBLUP” (Endelman, 2011) and “BGLR” (Pérez and de los Campos, 2014) containing the GS models rrBLUP and Bayesian Lasso (BL), respectively, were run in R software.

Prediction accuracy of seed protein was evaluated for different SNP sets, including 22 significant SNPs detected from GWAS, 22 random SNPs, 100 random SNPs, 250 random SNPs, 500 random SNPs, 1,000 random SNPs, 2,000 random SNPs, 5,000 random SNPs, and 10,115 SNPs. The effect of training population size on GS accuracy was investigated by conducting cross-validation at different levels with 100 replications for each cross-validation fold from two to ten.

## Results

### Seed Protein Content Variations in Two Populations

The seed protein content of the 180 RILs showed a biased normal distribution, seed protein content ranged from 34.69 to 58.71, and the Coefficient of variation (CV) was 23.39% (Supplementary Figure 2A). The seed protein content of the 284 accessions showed a biased normal distribution, seed protein ranged from 35.65 to 50.99, and the CV was 9.53% (Supplementary Figure 2B).

### Genetic Map Construction and Quantitative Trait Locus Mapping in Recombinant Inbred Line Population

The RIL population was genotyped by sequencing. After filtering, a total of 2,498 polymorphic markers SNP were obtained and were mapped to 20 soybean chromosomes, thus the genetic maps were built for the RILs (Supplementary Figure 3A). According to their physical positions in the genome assembly, these markers were basically evenly distributed on 20 chromosomes. The 20 combined maps between physical distance and genetic position showed a good match (Supplementary Figure 3B). Chr. 14 had the least number of markers (68) and Chr. 18 had the largest number of markers (184). A genetic linkage map with a total length of 4,476.2 cm was constructed and the average distance between two adjacent markers was 1.8 cm (Supplementary Figure 3). The average distance between adjacent markers was the smallest on Chr. 20 (1.32 cm) but was the largest on Chr. 9 (2.26 cm).

**FIGURE 1:**
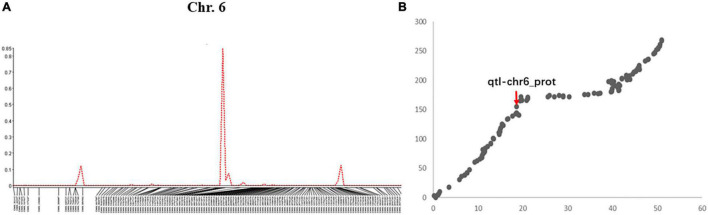
**(A)** QTL mapping of seed protein content in soybean chromosome 6 based on single-trait multiple IM (SMIM) in Qgene, **(B)** The QTL, qtl-chr6_prot was mapped on the combined map between physical distance and genetic position of the chromosome 6, where the x-axis shows physical distance (Mbp) and the y-axis shows the genetic position (cM).

A total of 5 QTLs on chromosomes 6, 8, 15, 17, and 20 were detected and the LOD value of the markers associated with the QTL ranged from 3.3 to 14.1; the QTL could explain 6.6%–29.6% of the genetic variation (Figure 1A and Supplementary Figure 4). Among these, one QTL with a positive allelic effect was from Jidou12 and 4 QTL with positive alleles were from Ye9 (Table 1). The QTL *qtl-chr6-prot* had the highest LOD and could explain 22.3–29.6% of genetic variation (Table 1 and Figure 1A). The *qtl-chr6-prot* was in the heterochromatic region (Figure 1B).

**TABLE 1:** Single nucleotide polymorphism (SNP) markers/quantitative trait locus (QTL) detected in recombinant inbred line (RIL) and natural populations.

SNP Markers/QTL Detected in RIL and Natural populations	Population	Model	Confidence interval	Physical position bp	LOD	Posterior (POP)	PVE (%)
qtl-chr6_prot	RIL	Bayesian IM	142	18864382		0.847	
		Single-trait multiple IM (SMIM)	146–152	18580363–18597849	11.46		25.40
		Single-trait CIM MLE (SMLE)	142–152	18580363–18864382	13.1		
		Single marker regression (SMR)	141.9–144.5	18449510–19398117	13.7		29.60
		ICIM	144	18449510–18597849	14.11		22.30
Chr6_18658898	POP	MLM		18658898	19.95		
		GLM		18658898	25.76		
qtl-chr8_prot	RIL	Bayesian IM	56–58	9318625–9502316		0.392–0.49	
		Single-trait multiple IM(SMIM)	42–44	7270752–8285888	7.16		16.70
		Single-trait CIM MLE (SMLE)	42–44	7270752–8285888	7.05		
		Single marker regression	41.6–45.6	7270752–8285888	6.79		16.10
		ICIM	61	9701254–9877332	6.35		9.18
qtl-chr15_prot	RIL	Bayesian IM	12	1890050		0.179	
			32	4708800–4708818		0.759	
			42	5786875		0.119	
		Single-trait multiple IM (SMIM)	20–32	3303648–4708818	3.28		8.00
		Single-trait CIM MLE (SMLE)	18–20	3380704–3303648	4.43		
			30–50	4708800–6651199	4.93		
		Single marker regression	14.2–19.7	2095208–3303648	4.57		11.00
			27.7–31.8	4370908–4708800	3.91		9.50
			45.1–54.5	6037184–7193889	4.43		10.70
		ICIM	20	3303648–3488588	4.72		6.60
qtl-chr17_prot1	RIL	Bayesian IM	100	12398690–12801544		0.941	
		Single-trait multiple IM (SMIM)	100–124	12398690–13632893	4.11		9.80
		Single-trait CIM MLE (SMLE)	104–112	12801549–13813134	4.05		9.90
		Single marker regression	99.5–103.9	12398690–12801549	4.4		
qtl-chr20_prot	RIL	Bayesian IM	112	33202705		0.871	
		Single-trait multiple IM (SMIM)	94	33202705	6.31		14.90
		Single-trait CIM MLE (SMLE)	86–114	26572911–33224754	5.34		
		Single marker regression	93–115.3	26572981–33507017	5.12		12.30
		ICIM	97	26957096–27003724	7.16		10.22
Chr20_34423091	POP	MLM		34423091	7.21		
Chr20_34423091		GLM		34423091	6.55		

### Genome-Wide Association Study in Natural Population and Candidate Genes Selection

**FIGURE 2:**
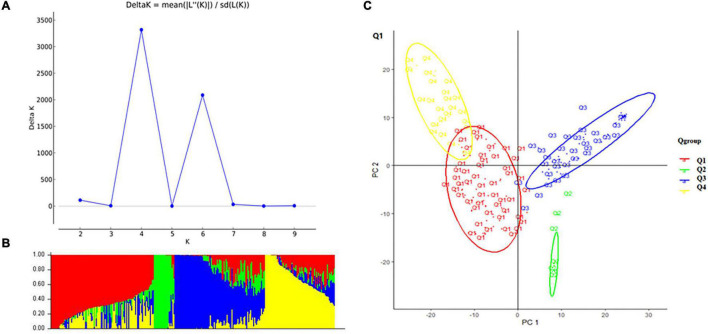
Structure analysis: **(A)** delta *K*-values for different numbers of populations (K) from the STRUCTURE analysis, the *x*-axis shows different numbers of populations (K), the *y*-axis shows delta *K*-values for different numbers of subpopulations (K). **(B)** Classification of 284 accessions into four subpopulations using STRUCTURE version 2.3.4, where the *x*-axis shows accessions and the *y*-axis shows the probability (from 0 to 1) of each accession belonging to subpopulation (Q = K) membership. The membership of each accession belonging to subpopulations is indicated by different colors (Q1, red; Q2, green; Q3, blue; and Q4, yellow). **(C)** Principal component analysis (PCA) of the population structure. Distribution of the accessions in the association panel under PC1 and PC2.

A total of 10,115 high-quality SNPs were used to perform population structure analysis of the 284 accessions using the STRUCTURE software (Kaeuffer et al., 2007). When *K* = 4, delta K was maximal with a relatively stable α value (Figures 2A,B). Cluster I was comprised of 102 accessions, including 77 cultivars, 21 landrace, and 5 exotic accessions; cluster II was comprised of 19 accessions, namely, 18 exotic accessions and 1 cultivar; cluster III was comprised of 93 accessions, namely, 57 exotic accessions, 34 cultivars, and 2 landraces; and cluster IV comprised of 70 accessions, namely, 51 landraces, 16 cultivars, and 3 exotic accessions. Principal component analysis (PCA) also showed the four groups (Figure 2C).

A significant association (-log *P* > 5.35) with seed protein was observed for 22 SNPs from 22 haplotype blocks in 13 of the 20 chromosomes using GLM and MLM (Table 2). The LOD of the 22 markers ranged from 6.6 to 20.1 in GLM analysis and 6.3 to 26.3 in MLM analysis (Table 2 and Supplementary Figure 5), indicating that these markers were strongly associated with seed protein. Eighteen of these markers were in euchromatic regions and four of these markers were in heterochromatic regions (Table 2).

**TABLE 2:** Significant SNPs associated with protein content over 4 years, chromosome (Chr.) and physical position (bp) of the significant SNPs, logarithm of odds (LOD) [-log10 (*p-*value)] values of generalized linear model (GLM) and mixed liner model (MLM), and allele with positive effect at the SNP locus.

SNP Markers	Chr.	Position	Heterochromatic region	Euchromatic region	SNP Type	Allele with positive effect	LOD of GLM	LOD of MLM	SNP annotation
Chr03_34851073	3	34,851,073		E	A/C	C	12.79	13.69	Glyma.03G133300
Chr03_42692363	3	42,692,363		E	C/T	C	10.22	9.18	Glyma.03G224600
Chr05_40074496	5	40,074,496		E	A/T	T	20.03	25.86	Glyma.05G221300
Chr05_41114434	5	41,114,434		E	C/T	C	13.08	13.12	Upstream_gene_variant| MODIFIER| Glyma.05G234000
Chr06_14606307	6	14,606,307		E	A/G	G	9.30	8.41	Upstream_gene_variant| MODIFIER| Glyma.06G173600
Chr06_18658898	6	18,658,898	H		A/G	A	19.95	25.76	Glyma.06G202000
Chr08_10757609	8	10,757,609		E	C/T	C	7.23	6.65	Glyma.08G140700
Chr09_5898756	9	5,898,756		E	A/G	G	8.80	8.45	Glyma.09G062100
Chr09_45699847	9	45,699,847		E	A/G	G	8.55	7.64	Glyma.09G234500
Chr10_2992389	10	2,992,389		E	A/T	A	8.47	7.94	Glyma.10G034400
Chr10_44549078	10	44,549,078		E	A/G	G	9.22	8.80	Glyma.10G213000
Chr12_1536444	12	1,536,444		E	A/G	G	7.48	6.29	Glyma.12G021400
Chr14_2351357	14	2,351,357		E	C/T	C	10.58	9.26	Glyma.14G032300
Chr14_48312781	14	48,312,781		E	C/G	G	20.07	26.26	Upstream_gene_variant| MODIFIER| Glyma.14G218000
Chr15_13541492	15	13,541,492	H		C/G	C	8.48	7.71	Upstream_gene_variant| MODIFIER| Glyma.15G160000
Chr17_347445	17	347,445		E	A/T	A	9.38	9.40	Glyma.17G003000
Chr17_32480031	17	32,480,031	H		A/G	G	6.64	6.73	Intergenic_region| MODIFIER| Glyma.17G203300-Glyma.17G203400
Chr18_7837981	18	7,837,981		E	A/C	C	14.27	13.62	Glyma.18G081200
Chr18_18834295	18	18,834,295		E	A/C	C	8.24	7.25	Intergenic_region| MODIFIER| Glyma.18G133000-Glyma.18G133100
Chr18_50849168	18	50,849,168		E	A/T	A	11.29	11.66	Upstream_gene_variant| MODIFIER| Glyma.18G221300
Chr19_12210884	19	12,210,884	H		C/T	T	19.91	25.75	Intergenic_region| MODIFIER| Glyma.19G060900-Glyma.19G061000
Chr20_34423091	20	34,423,091		E	A/T	T	7.21	6.55	Glyma.20G100900

Two significant SNP loci on Chr. 6 and 20 were detected in linkage analysis and GWAS and the SNP loci detected on Chr. 6 by GWAS were in the QTL intervals obtained by linkage analysis. This SNP region on Chr. 6 had a high Phenotypic variation explained (PVE) (22.3–29.60%) and LOD (6.696–25.762). The region on Chr. 20 was associated with protein content with a PVE of 12.30% and LOD of 7.208 (Tables 1, 2).

**FIGURE 3:**
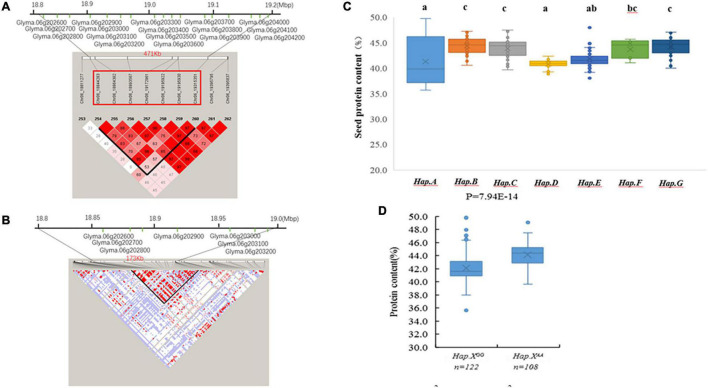
**(A)** The extent of linkage disequilibrium (LD) in the regions based on pairwise *r*^2^ values. The *r*^2^ values are indicated using the color intensity index. Heatmap showing LD between each pair of markers that passed the Bonferroni threshold in genome-wide association study (GWAS). **(B)** Candidate genes for each single nucleotide polymorphism (SNP) locus. The bottom panel depicts the extent of linkage disequilibrium in the regions based on pairwise *r*^2^ values. The *r*^2^ values are indicated using the color intensity index shown. **(C)** Boxplot of seed protein based on different genotypes in soybean accessions. **(D)** Boxplot of seed protein based on Hap.X*^GG^* and Hap.X*^AA^* phenotypic differences between genotype combinations of the two SNPs.

A 471-kb haplotype block from Chr6_18844283 to Chr6_19315351 included 7 SNP markers and 17 genes (Figure 3A). Pairwise LD analysis of the imputed SNP data showed that the candidate gene region was from Chr6_18842491 bp to Chr6_19015855 bp (Figure 3B). Seven candidate genes were in the regions, which included polynucleotidyl transferase (Glyma.06G202900 and Glyma.06G203100), polygalacturonase activity (Glyma.06G202600 and Glyma.06G203000), ATP synthase (Glyma.06G203200), and genes without annotation (Glyma.06G202700 and Glyma.06G202800) (Figure 3B).

There were 7 QTL haplotypes in the LD block from Chr6_18844283 to Chr6_19315351 in the natural population that showed differences in protein content (Supplementary Table 2 and Figure 3C). The haplotypes Hap.B, Hap.C, and Hap.F had higher protein content than other haplotypes. Hap.B had the highest protein content, but no significant difference was observed among Hap.B, Hap.C, Hap.F, and Hap.G (Figure 3C). Further analysis showed that the SNP located at Chr6_19172961 may be more important; varieties carrying Hap.X*^AA^* showed higher protein content than Hap.X*^GG^* (Figure 3D).

### Prediction Accuracy of Seed Protein Content

**FIGURE 4:**
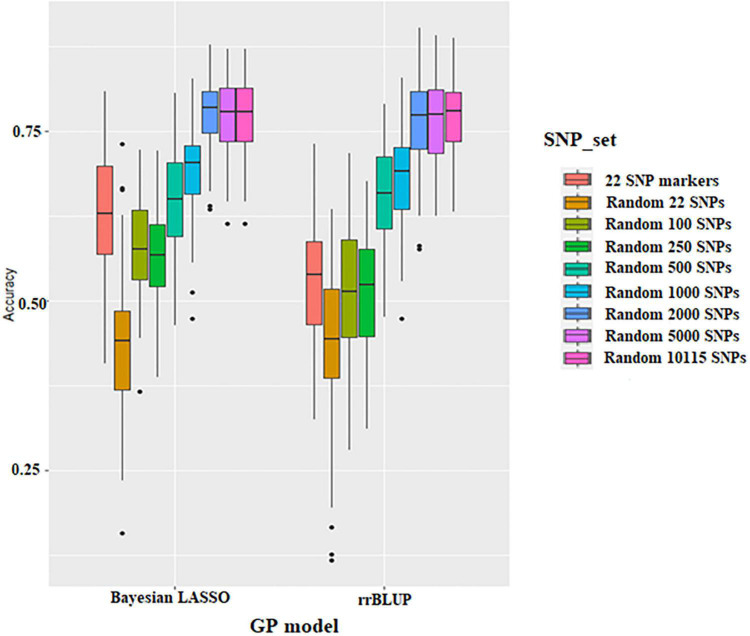
Boxplots show the effect of different SNP density sets on genomic selection in the Bayesian Lasso Regression (BLR) model and ridge regression best linear unbiased prediction (rrBLUP) models.

Prediction accuracy of different SNP densities for seed protein was conducted using 22 significant SNPs resulting from GWAS and 22 to 10,115 random SNPs, respectively. The prediction accuracy ranges from 0.44 to 0.77 using the rrBLUP model and from 0.44 to 0.78 using the BLR model (Figure 4 and Supplementary Table 3). BLR and rrBLUP performed similarly for prediction accuracy; the average prediction accuracy was 0.63 and 0.53, respectively. The prediction accuracy of the 22 SNPs obtained from GWAS was higher than that of random 22 SNPs and random 250 SNPs (Figure 4 and Supplementary Table 3). Thus, regardless of the GS model, the accuracy of GS was higher when the significant SNPs from GWAS were used. Prediction accuracy for seed protein was increased with higher SNP density. However, there is a minimal difference in prediction accuracy after the SNP number reached 2,000 (Figure 4 and Supplementary Table 3).

**FIGURE 5:**
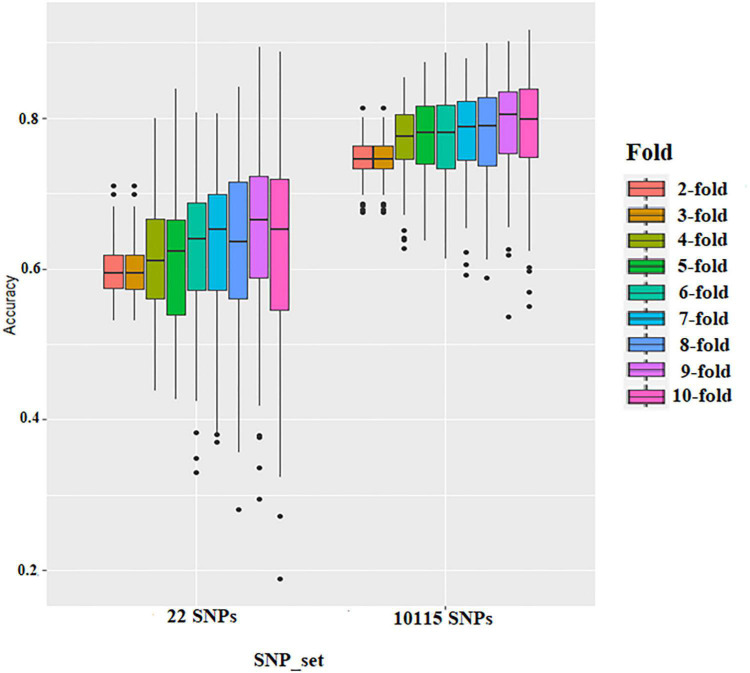
Boxplots show the effect of training population size on genomic selection accuracy by conducting cross-validation at different folds with 100 replications for each cross-validation fold using rrBLUP.

The effect of training population size on GS accuracy was also investigated by conducting cross-validation at different folds with 100 replications for each cross-validation (Figure 5 and Supplementary Tables 4, 5). On average, the prediction accuracy of the BLR model was 0.62 using GWAS-derived SNPs and 0.77 using the whole set of SNPs (Figure 5 and Supplementary Table 4). The prediction accuracy of rrBLUP was less than BLR, with 0.5 using GWAS-derived SNPs and 0.77 using the whole set of SNPs (Figure 5 and Supplementary Table 5). Considering average *r*-value and standardized deviation Sn, sevenfold resulted in a high *r*-value and low Sn in BLR models and sixfold resulted in a high *r*-value and low Sn in rrBLUP models.

## Discussion

### Quantitative Trait Locus Mapping and Candidate Genes Identification for Soybean Seed Protein

Wild soybean with desired traits may improve the yield, quality, and other traits of cultivated soybeans. In this study, we performed QTL mapping for protein content in a RIL population derived from the cross of cultivated Jidou12 and wild soybean Ye9. Five major stable QTLs were detected on Chr. 6, 8, 15, 17, and 20 using Bayesian IM, SMIM, SMLE, and SMR models in Q-gene and IciMapping. Among these QTLs, we discovered that *qtl-chr6_prot* contributed an average of 25.77 of the phenotypic variance and the positive additive effects of allele were from the cultivated soybean Jidou12. The *qtl-chr6_prot* did not overlap with or was not adjacent to any of the previously reported QTLs for seed protein content. Other QTLs, *qtl-chr8_prot, qtl-chr15_prot, qtl-chr17_prot*, and *qtl-chr20_prot*, explained an average of 13.99, 9.1, 9.85, and 12.47 of the phenotypic variance, respectively; the positive additive effects of the allele of these QTL were from the wild soybean parent. The QTL *qtl-chr8_prot* (7.27–8.29 Mb) overlapped with the QTLs, as previously reported by Pathan et al. (2013). In addition, the QTL *qtl-chr15_prot* (3.30–4.71 Mb) overlapped with the *qPro15-1* (Zhang et al., 2019) and *qtl-chr17_prot* (12.80–13.81 Mb) with the protein 26-2 (Reinprecht et al., 2006). The position of QTL *qtl-chr20_prot* (26.57–33.51 Mb) was consistent with that of the confirmed QTL *cqPro-20* (Diers et al., 1992; Pandurangan et al., 2012; Vaughn et al., 2014; Sonah et al., 2015; Warrington et al., 2015; Zhang Y. et al., 2018; Fliege et al., 2022). Fliege et al. (2022) concluded that a transposon insertion within the CONSTANS, CO-like, and TOC1 (CCT) domain protein encoded by the *Glyma.20G85100* gene accounted for the high/low seed protein alleles of the cqSeed protein-003 QTL (31.74–31.84 Mb).

In the novel QTL region, the *qtl-chr6_prot*, seven candidate genes were identified. Of which, Glyma06G202900 and Glyma06G203100 were annotated as polynucleotidyl transferase, ribonuclease H-like superfamily protein, which were homologous to the AT5G61090 gene in *Arabidopsis*. The protein encoded by the AT5G61090 had an RNA–DNA hybrid ribonuclease activity (Stoppel and Meurer, 2012). Glyma06G202600 was annotated as plasmodesmata callose-binding protein 3, homologous to AT1G18650 with callose-binding activity and the regulating intercellular trafficking in *Arabidopsis* (Simpson et al., 2009). Glyma06G203000 was annotated as a pectin lyase-like superfamily protein homologous to AT3G07820 with a polygalacturonase activity in *Arabidopsis* (Kim et al., 2006). Glyma06G203200 was annotated as a gamma subunit of Mt ATP synthase, homologous to AT2G33040, one of mitochondrial (mt) ATP synthesis subunits. Reduced expression of these subunits of the mt ATP synthase was proposed to disturb cellular redox states (Robison et al., 2009).

### Genomic Selection in Soybean

Genomic selection overcomes the problems of traditional breeding methods and MAS selection and provides a new way for the selection of quantitative traits controlled by genes with minor effects. GS allows for the estimation of the effects of all the markers across the genome. These effects can be used to predict the performance of lines (Meuwissen et al., 2001). Since the target trait phenotype of an individual is predicted using the GS model, the materials could be screened and selected before planting, thus reducing costs and improving breeding efficiency (Heslot et al., 2012; Longin et al., 2015; Spindel et al., 2015). Matei et al. (2018) showed that the selection cycle for yield and seed weight can be significantly shortened using GS.

So far, the GS study has been mainly conducted on maize, wheat, and rice. The GS study in soybean remains limited. In 2013, Shu performed GS for 100-seed weight and reported a prediction accuracy of 0.904 (Shu et al., 2013). Subsequent GS showed accuracy for soybean cyst nematode (SCN) was 0.59–0.67 (Bao et al., 2014) and 0.64 for soybean yield (Jarquín et al., 2014).

The GS was performed on amino acid concentration (Qin et al., 2019), soybean chlorophyll content, soybean cyst nematode tolerance (Ravelombola et al., 2019), yield, and yield-related traits, such as maturity, plant height, and 100-seed weight (Ravelombola et al., 2021). These studies have shown the feasibility of GS for soybean yield and quality-related traits (Matei et al., 2018; Stewart-Brown et al., 2019).

However, few reports have focused on the GS of seed protein in soybean. Stewart-Brown et al. (2019) evaluated the potential of GS for soybean seed protein using 483 elite breeding lines from 26 biparentals and reported the predictive abilities of 0.81 in all the populations, 0.55 across populations, and 0.60 within each biparental population. Duhnen et al. (2017) compared genomic prediction accuracy of seed protein obtained using models calibrated across or within two subpopulations: early lines and late lines. The results showed that calibrations within subpopulations were more efficient. Five Bayesian models were also compared with Genomic best linear unbiased prediction (GBLUP) and did not show improved prediction accuracy. In this study, we performed GS based on different SNP sets, different training population sizes, and statistical models. The results showed that the use of GWAS-derived SNPs for conducting GS significantly improved the accuracy of prediction, which was consistent with the results reported by Qin et al. (2019). The model selection criteria, SNP sets, and population training size were critical factors when conducting a GS, as reported in previous studies (Ravelombola et al., 2019, 2020, 2021). Those studies had demonstrated that 1,000–2,000 genome-wide markers across all the lines/accessions were needed to reach maximum efficiency of genomic prediction in the populations, increasing marker density that would not improve prediction efficiency (Poland et al., 2012; Bao et al., 2014; Zhang J. et al., 2016; Song et al., 2020). This study showed that there was a minimal difference in prediction accuracy after the SNP number reached 2,000 for seed protein content.

## Conclusion

This study reported mapping and GS for seed protein content. Molecular markers associated with seed protein content were identified in RIL and natural populations and a novel QTL for seed protein content was detected and mapped on Chr. 6 in both populations. In addition, seven candidate genes that were related to seed protein content were identified. This is one of a few reports investigating seed protein content using RILs derived from cultivated and wild soybean crosses. Our results showed that GS accuracy was dependent on the SNP set and training population size; a set of GWAS-derived SNPs could increase GS accuracy. No significant GS accuracy difference was observed between rrBLUP and BL models. The results demonstrated the potential of using GS to improve soybean seed protein content.

## Data Availability Statement

The datasets presented in this study can be found in online repositories. The names of the repository/repositories and accession number(s) can be found in the article/Supplementary Material.

## Author Contributions

JQ and AS: data curation. JQ, CY, and LY: funding acquisition. FW, QZ, and TZ: investigation. JQ, AS, and WR: methodology. MZ, LY, and CY: project administration. AS: software. FW and TZ: validation. JQ: writing – original draft preparation. JQ, QS, and AS: writing - review and editing. All authors contributed to the article and approved the submitted version.

## Conflict of Interest

The authors declare that the research was conducted in the absence of any commercial or financial relationships that could be construed as a potential conflict of interest.

## Publisher’s Note

All claims expressed in this article are solely those of the authors and do not necessarily represent those of their affiliated organizations, or those of the publisher, the editors and the reviewers. Any product that may be evaluated in this article, or claim that may be made by its manufacturer, is not guaranteed or endorsed by the publisher.

## Footnotes

1
https://www.soybase.org/

2
https://www.arabidopsis.org/
